# Sacubitril/valsartan (LCZ696) ameliorates hyperthyroid-induced cardiac hypertrophy in male rats through modulation of miR-377, let-7 b, autophagy, and fibrotic signaling pathways

**DOI:** 10.1038/s41598-022-18860-y

**Published:** 2022-08-27

**Authors:** Tarek Khamis, Amira Ebrahim Alsemeh, Doaa M. Abdullah

**Affiliations:** 1 Department of Pharmacology, Faculty of Veterinary Medicine, Zagazig University, Zagazig, 44519 Egypt; 2 Laboratory of Biotechnology, Faculty of Veterinary Medicine, Zagazig University, Zagazig, 44519 Egypt; 3 Human Anatomy and Embryology Department, Faculty of Medicine, Zagazig University, Zagazig, 44519 Egypt; 4 Clinical Pharmacology Department, Faculty of Medicine, Zagazig University, Zagazig, 44519 Egypt

**Keywords:** Biochemistry, Cell biology, Cardiology, Endocrinology

## Abstract

Hyperthyroidism is associated with cardiac hypertrophy, fibrosis, and increased risk of cardiovascular mortality. Sacubitril/valsartan (LCZ696) is a new combined drug that has shown promise for the treatment of hyperthyroidism-associated heart failure; however, the underlying molecular mechanisms, including the contributions of epigenetic regulation, remain unclear. The present study was designed to investigate the therapeutic efficacy of LCZ696 and the potential contributions of microRNA regulation in a rat model of hyperthyroidism-induced cardiac hypertrophy. Cardiac hypertrophy was induced by intraperitoneal administration of levothyroxine. Sixty adult male Wistar rats were randomly allocated to four equal groups (15 rats each): control, cardiac hypertrophy (CH), CH + valsartan, and CH + LCZ696. Treatment with LCZ696 or valsartan significantly improved hemodynamic abnormalities, normalized serum concentrations of natriuretic peptide, fibroblast growth factor-23, and cardiac inflammatory markers compared to CH group rats. Treatment with LCZ696 or valsartan also normalized myocardial expression levels of autophagy markers, fibrotic markers, *PPAR-ϒ, mir-377, and let-7b*. In addition, both valsartan and LCZ696 ameliorated collagen deposition, ventricular degeneration, and various ultrastructural abnormalities induced by levothyroxine. The beneficial effects of LCZ696 were superior to those of valsartan alone. The superior efficacy of LCZ696 may be explained by the stronger modulation of *miR-377* and *let-7b*.

## Introduction

Thyroid hormones (THs) are critical regulators of cellular metabolism, so chronic abnormal TH signaling has progressive and markedly deleterious effects on tissues and organs, especially in the cardiovascular system^1^. Hyperthyroidism predisposes the heart to both structural and functional abnormalities^2^, including cardiac hypertrophy and eventual heart failure^3^. This hyperthyroid-induced cardiac hypertrophy is triggered by both the direct actions of THs on myocardial cells and indirect actions through effects on other endocrine systems such as the sympathetic nervous system (SNS) and renin-angiotensin system (RAS) amongst others^1^. Thyroid hormone stimulates both local (cardiac) and peripheral renin-angiotensin systems^3^, and cardiac hypertrophy can be suppressed by inhibitors of angiotensin-converting enzyme and angiotensin type II receptors^4^. For instance, angiotensin II type 1 (AT1) receptor blockers such as valsartan are used clinically to suppress cardiac remodeling due to hyperthyroidism^5^.

Several potential mechanisms have been proposed to explain TH-induced cardiac hypertrophy, including dysregulation of microRNAs that normally serve to modulate the expression of genes involved in pathogenic or reparative processes^6^. Among potential miRNA targets of TH, miR-377 was recently implicated in the onset of cardiac hypertrophy through inhibition of autophagy^7^. In addition, the miRNA *let-b7* was reported to ameliorate cardiac hypertrophy and fibrosis via downstream regulation of TGF-β/SMAD pathway gene expression^8^. Autophagy is a quality control system that serves to recycle the basic constituents of damaged macromolecules and organelles for energy conservation and nutrient preservation under stress^9^. In hyperthyroidism, autophagy is vital for cardiac remodeling and conservation of function^10^.

Inflammatory response and oxidative stress pathways have also been implicated in the development of hyperthyroidism-induced cardiac hypertrophy^4^. Araujo et al.^11^ found that triiodothyronine (T3)-induced cardiac hypertrophy was associated with accelerated mitochondrial free radical production and reduced cellular antioxidant capacity in cardiomyocytes, resulting in reactive oxygen species accumulation and oxidative stress. THs also enhance the expression of inflammatory cytokines, triggering immune responses that may act synergistically with oxidative stress to induce cardiac remodeling and functional damage^12^. Upregulation of vasoactive peptides, especially cardiac natriuretic peptides (ANP and BNP), is a promising therapeutic strategy against cardiovascular diseases. Natriuretic peptides (NPs) stimulate vasodilatation and diuresis, thereby lowering blood pressure, favoring myocardial relaxation, and decreasing left ventricular hypertrophy^13^. Neprilysin is the key enzyme implicated in the degradation of natriuretic peptides NPs, and inhibition of this enzyme has been shown to reduce inflammation, oxidative stress, and fibrosis in a model of myocardial hypertrophy^14^. Sacubitril/valsartan (LCZ696) is a fixed-dose combination of the AT1R blocker valsartan and the neprilysin inhibitor sacubitril (termed an angiotensin II receptor-neprilysin inhibitor or ARNI)^15^ shown to augment the beneficial effects of BNP while preventing the unwanted harmful effects of RAS activation (inflammation, tissue damage, and fibrosis). Numerous studies have reported that LCZ696 can enhance cardiac function and diminish cardiac hypertrophy and fibrosis in various models of heart failure^14,15^. However, the exact molecular mechanisms, including the contributions of epigenetic regulation, are largely unknown.

The present study was designed to investigate the therapeutic efficacy of valsartan and/or sucabitril in a rat model of hyperthyroidism-induced cardiac hypertrophy and elucidate the contributions of *miR-377*, *let-7b*, and the molecular pathways regulated by these miRNAs.

## Results

### Validation of experimental hyperthyroidism and cardiac hypertrophy

**Figure 1:**
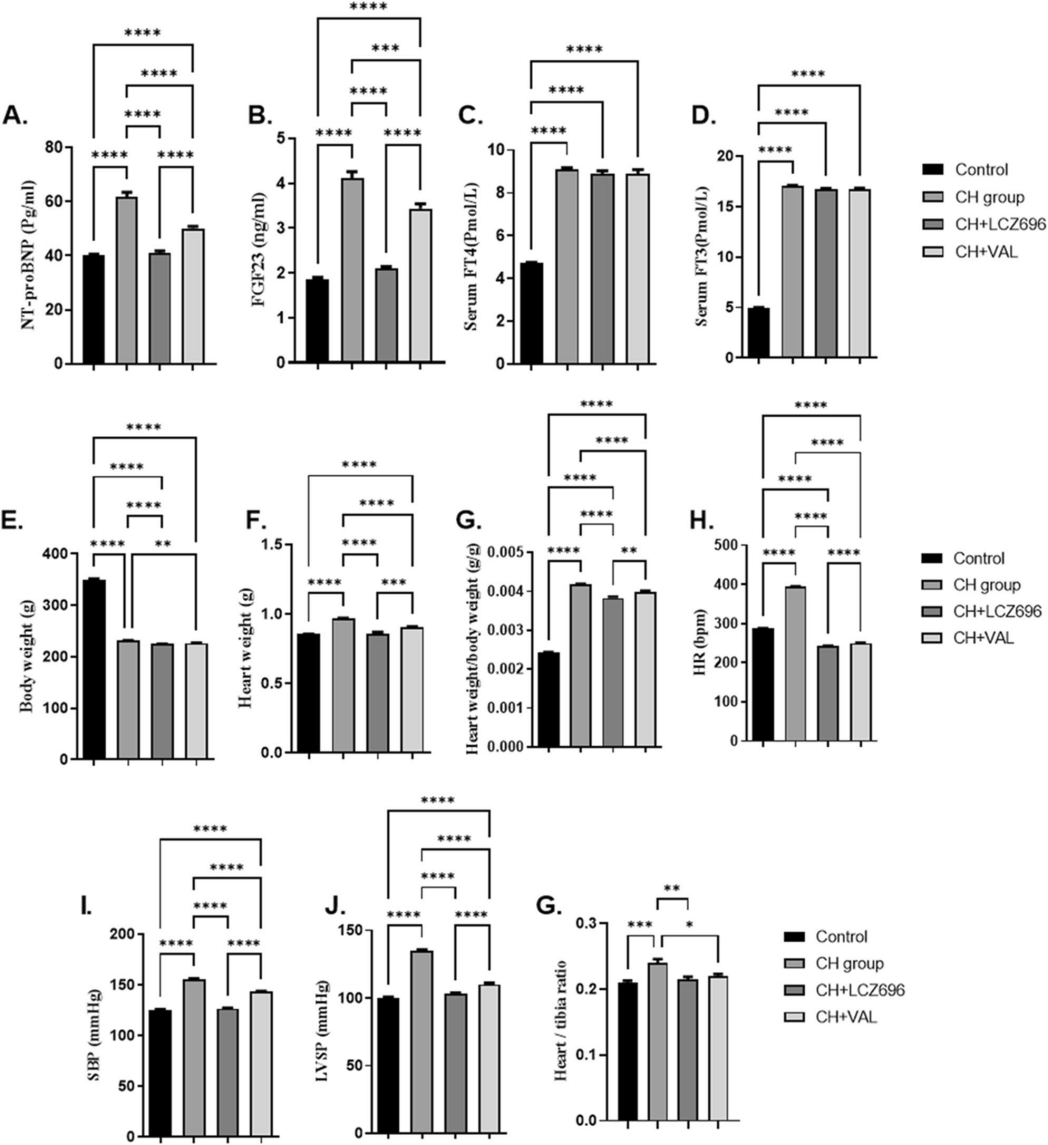
The effect of the LCZ696 and VAL administration on the hemodynamic parameters, cardiac hypertrophy markers, serum thyroid hormonal level (**A**–**J**). (**A**) serum NT-proBNP level (Pg/mL). (**B**) serum FGF23 level (ng/mL), (**C**) serum FT4 level (Pmol/L), (**D**) serumFT3 level (Pmol/L), (**E**) body weight (g), (**F**) heart weight (g), (**G**) heart/body weight ratio, (**H**) heart rate (HR) bpm, (**I**) systolic blood pressure (SBP) mmHg, (**J**) left ventricular systolic pressure (LVSP) mmHg, and (**K**) heart weight/tibia ratio. Values are mean of 6 rats per group ± S.E.M *p < 0.05; **p < 0.01; ***p < 0.001. Group means were compared by one-way analysis of variance (ANOVA) followed by Tukey post hoc tests for multiple comparisons.

To confirm the hyperthyroid status and cardiac hypertrophy of model rats, serum free T3 and T4 concentrations, hemodynamic parameters heart rate(HR), systolic blood pressure (SBP), and left ventricular systolic pressure (LVSP), and serum levels of the cardiac hypertrophy markers NT-proBNP and FGF-23 were measured (Fig. 1A–J) after the final day of treatment. Consistent with successful modeling, free T3, free T4, SBP, HR, and LVSP were significantly higher in T4-treated cardiac hypertrophy (CH group) rats than in vehicle-treated control animals (all *p* < 0.001) (Fig. 1C, D, H, I, J). Moreover, the HW/BW ratio was also significantly higher in CH group rats than controls (*p* < 0.05) (Fig. 1E–G), as were serum concentrations of the cardiac hypertrophy markers NT-pro BNP and FGF-23 (both *p* < 0.001) (Fig. 1A, B).

### LCZ696 and VAL reduced serum markers of cardiac hypertrophy in hyperthyroid model rats

Treatment with VAL or LCZ significantly reduced all the aforementioned indices of hyperthyroidism-induced cardiac hypertrophy (all *p* < 0.001), although these reductions were consistently larger in the LCZ696 group (Fig. 1A–J).

### LCZ696 and VAL reversed inflammatory and autophagic marker changes in hyperthyroid model rats

**Figure 2:**
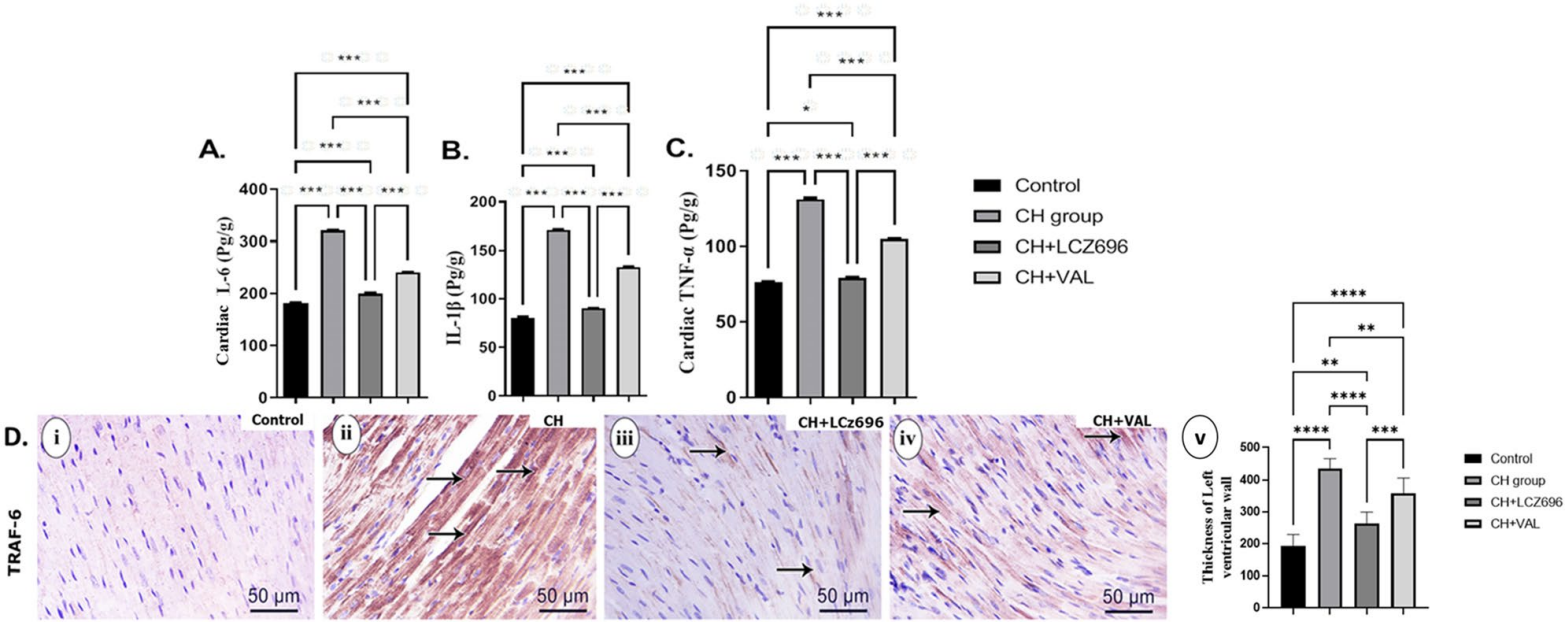
The effect of the LCZ696 and VAL administration on the level of the cardiac inflammatory markers (**A**–**D**). (**A**) cardiac IL-6 level (Pg/g), (**B**) cardiac IL-1β level (Pg/g), (**C**) cardiac TNF-α (Pg/g) level, (**D**) (i–v) representative photomicrographs showing the estimation of immunohistochemical staining of TRAF-6 in rat cardiac tissues of the different experimental groups (i) Control group, (ii) CH group, (iii) CH + LCZ696 group, (iv) CH + VAL group, and (v) a bar chart demonstrates the changes in the area % of TRAF-6 immune-positive cells of the different experimental groups. Values are mean of 6 rats per group ± S.E.M *p < 0.05; **p < 0.01; ***p < 0.001. Group means were compared by one-way analysis of variance (ANOVA) followed by Tukey post hoc tests for multiple comparisons.

The CH group exhibited significantly higher serum concentrations of the pro-inflammatory cytokines IL-6, IL-1 β, and TNF- α compared to Control group rats, and all of these increases were partially reversed by VAL and more strongly reversed by LCZ696 (Fig. 2A–C). Furthermore, immunohistochemical expression of TRAF6 protein in the LV was significantly elevated in the CH group compared to the Control group (*p* < 0.001) (Fig. 2A–C) and significantly downregulated by LCZ696 and VAL compared to the CH group (*p* < 0.001) (Fig. 2A–C). Again, the reduction was larger in the CH + LCZ696 group than the CH + VAL group (*p* < 0.01) (Fig. 2D).

**Figure 3:**
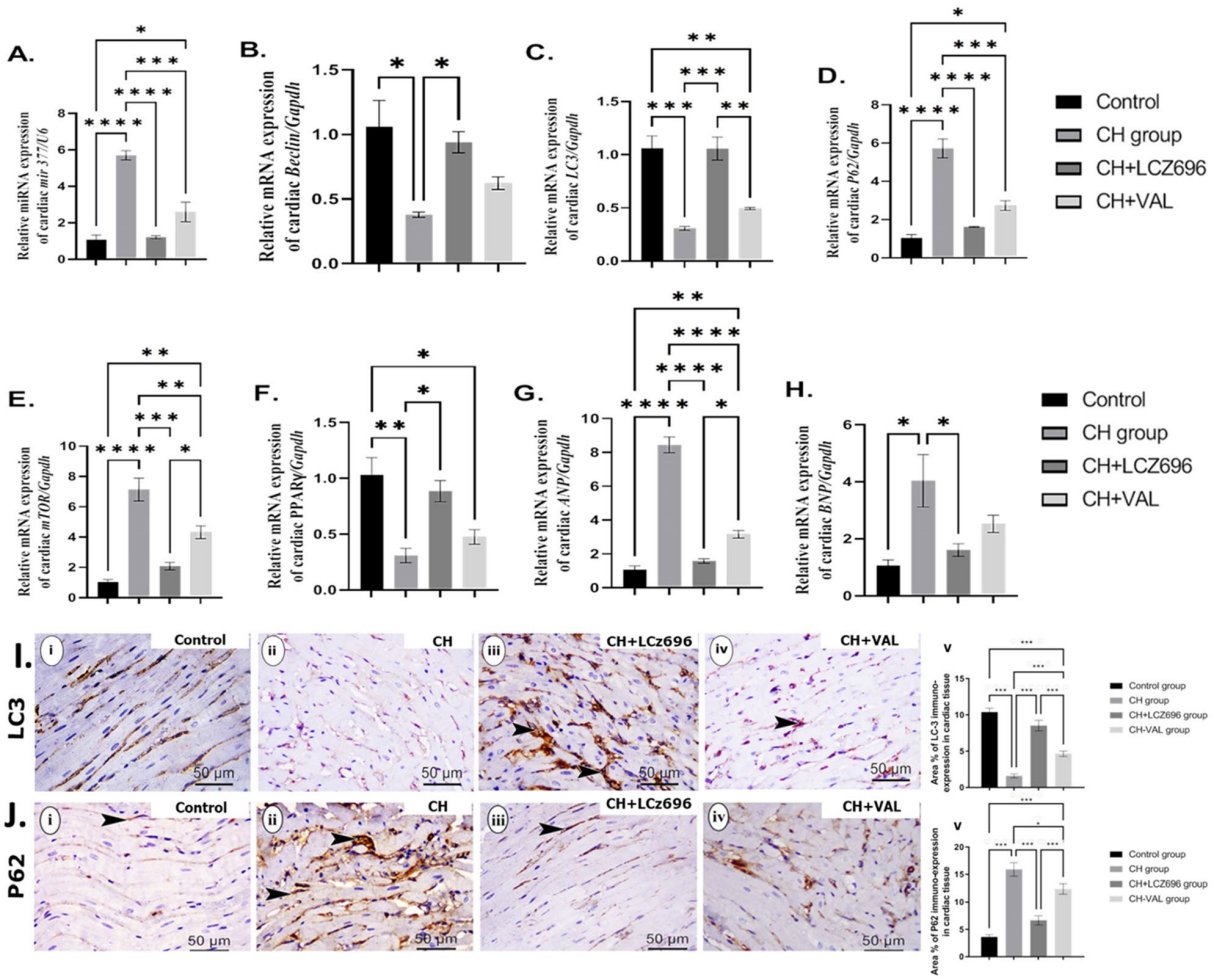
The effect of the LCZ696 and VAL administration on the expression of the cardiac autophagy, natriuretic-peptide, and their regulatory miRNA (**A**–**J**). (**A**) relative *miRNA* expression of cardiac *mir-377/U6* (%control), (**B**) relative *mRNA* expression of cardiac *Beclin/Gapdh* (%control), (**C**) relative *mRNA* expression of cardiac *LC-3/Gapdh* (%control), (**D**) relative *mRNA* expression of cardiac *P62/Gapdh* (%control), (**E**) relative *mRNA* expression of cardiac *mTOR/Gapdh* (%control), (**F**) relative *mRNA* expression of cardiac *PPAR-ϒ/Gapdh* (%control), (**G**) relative *mRNA* expression of cardiac *ANP/Gapdh* (%control), (**H**) relative *mRNA* expression of cardiac *BNP/Gapdh* (%control), (**I**) (i–v) representative photomicrographs showing the estimation of immunohistochemical staining of LC-3 in rat cardiac tissues of the different experimental groups (i) Control group, (ii) CH group, (iii) CH + LCZ696 group, (iv) CH + VAL group, and (v) a bar chart demonstrates the changes in the area % of LC-3 immune-positive cells of the different experimental groups, and (**J**) (i–v) representative photomicrographs showing the estimation of immunohistochemical staining of P62 in rat cardiac tissues of the different experimental groups (i) Control group, (ii) CH group, (iii) CH + LCZ696 group, (iv) CH + VAL group, and (v) a bar chart demonstrates the changes in the area % of P62 immune-positive cells of the different experimental groups. Values are mean of 6 rats per group ± S.E.M *p < 0.05; **p < 0.01; and ***p < 0.001. . Group means were compared by one-way analysis of variance (ANOVA) followed by Tukey post hoc tests for multiple comparisons.

The CH group also demonstrated significant upregulation of cardiac *miR-377, P62, mTOR, ANP*, and *BNP* expression levels as well as significant downregulation of *LC-3, Beclin*, and *PPAR-ϒ* expression levels compared to the Control group (all *p* < 0.001) (Fig. 3A–H) as measured by qPCR. All of these expression changes were reversed (normalized) by LCZ696 and by valsartan, with consistently stronger effects of LCZ696 compared to valsartan alone (Fig. 3A–H). Immunohistochemical staining of cardiac tissues from CH group rats revealed significant downregulation of LC-3 protein expression (*p* < 0.001) (Fig. 3I, ii) and significant upregulation of P62 protein expression (*p* < 0.001) compared to the Control group (Fig. 3I i, J i, respectively) as measured by % positive area. Again, treatment with LCZ696 significantly reversed both LC-3 downregulation (Fig. 3I, iii) and P62 protein upregulation (Fig. 3J, iii) compared to the CH group (*p* < 0.001). Treatment with VAL also significantly upregulated expression of LC-3 (Fig. 3I, iv) and significantly downregulated expression of P62 (Fig. 3J, iv) compared to the CH group (*p* < 0.01), but the magnitudes of these changes were smaller than induced by LCZ696.

### LCZ696 and VAL administration reversed the CH-associated changes in cardiac fibrotic markers and their regulatory miRNAs

**Figure 4:**
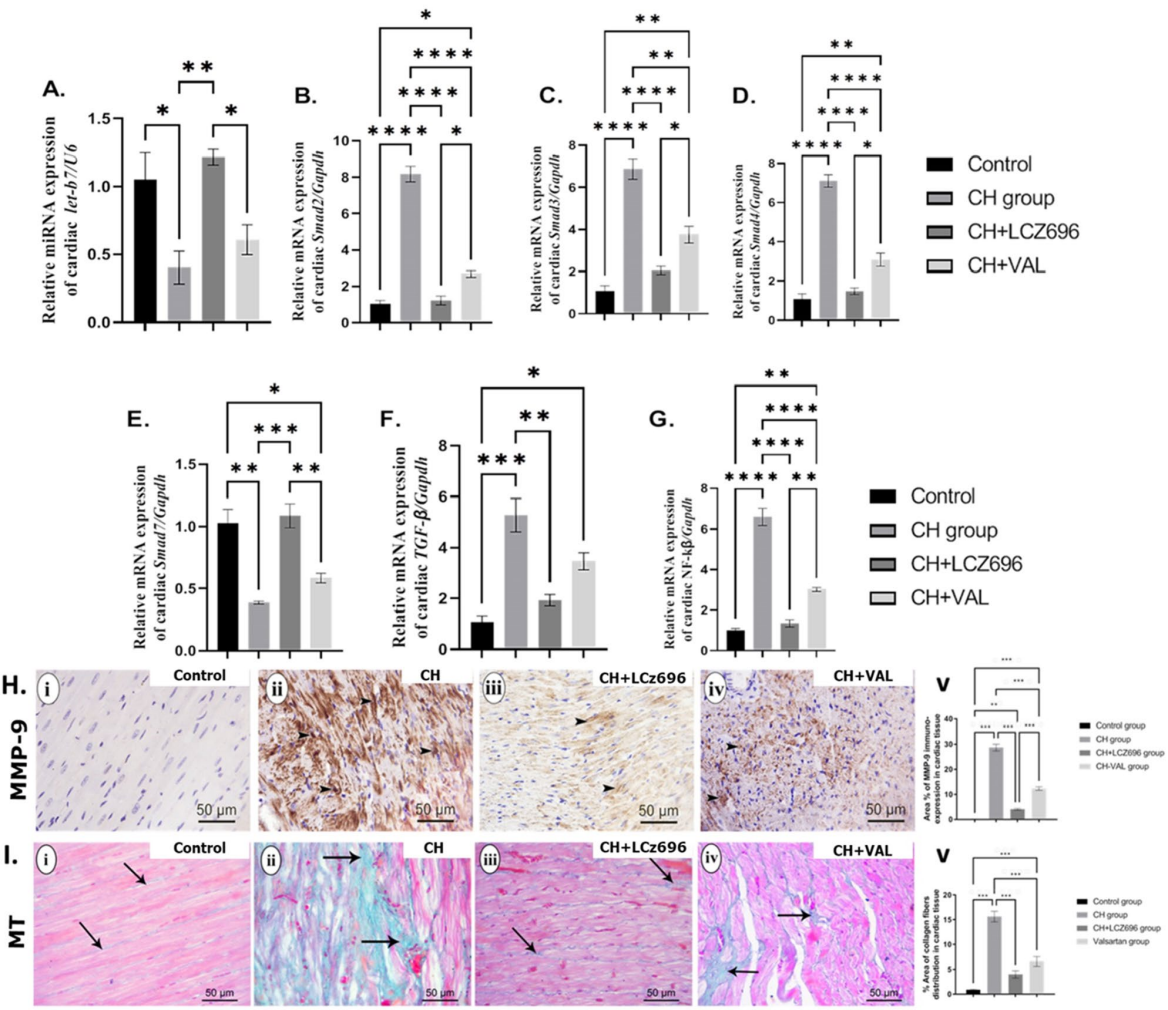
The effect of LCZ696 and VAL administration on the mRNA and protein expression of the cardiac fibrotic markers and their regulatory miRNA (**A**–**I**). (**A**) relative *miRNA* expression of cardiac *Let-7b/U6* (%control), (**B**) relative *mRNA* expression of cardiac *SMAD-2/Gapdh* (%control), (**C**) relative *mRNA* expression of cardiac *SMAD-3/Gapdh* (%control), (**D**) relative *mRNA* expression of cardiac *SMAD-4/Gapdh* (%control), (**E**) relative *mRNA* expression of cardiac *SMAD-7/Gapdh* (%control), (**F**) relative *mRNA* expression of cardiac *TGF-β/Gapdh* (%control), (**G**) relative *mRNA* expression of cardiac *NF-κβ/Gapdh* (%control), (**H**) (i–v) representative photomicrographs showing the estimation of immunohistochemical staining of MMP-9 in rat cardiac tissues of the different experimental groups (i) Control group, (ii) CH group, (iii) CH + LCZ696 group, (iv) CH + VAL group, and (v) a bar chart demonstrates the changes in the area % of MMP-9 immune-positive cells of the different experimental groups, and (**I**) (i–v) representative photomicrographs showing the estimation of the collagen deposition by Masson Trichome staining in rat cardiac tissues of the different experimental groups (i) Control group, (ii) CH group, (iii) CH + LCZ696 group, (iv) CH + VAL group, and (v) a bar chart demonstrates the changes in the area % of the collagen deposition of the different experimental groups. Values are mean of 6 rats per group ± S.E.M *p < 0.05; **p < 0.01; ***p < 0.001. Group means were compared by one-way analysis of variance (ANOVA) followed by Tukey post hoc tests for multiple comparisons.

Sections of LV from the CH group exhibited significant upregulation of the cardiac fibrotic marker genes *SMAD-2, SMAD-3, SMAD-4, TGF-β, and NF-κβ* as well as significant downregulation of *Let-b7* and *SMAD-7* compared to the Control group (all *p* < 0.001) (Fig. 4A–G). Consistent with antifibrotic activity, both LCZ696 and valsartan administration significantly reversed (normalized) all of these expression changes (*p* < 0.001), with greater effects of LCZ696 than valsartan alone (*p* < 0.01) (Fig. 4A–G).

Cardiac tissues from CH group rats also showed significantly greater immunohistochemical expression of MMP-9 (Fig. 4H, ii) compared to the Control group (*p* < 0.001) (Fig. 4H, i). Again consistent with robust antifibrotic activity, the upregulation of MMP-9 was significantly reversed (downregulated) by LCZ696 and by VAL compared to the CH group (*p* < 0.001) (Fig. 4H, iii), with a significantly stronger effect of LCZ696 than VAL (*p* < 0.01) (Fig. 4H, iv). Both LCZ696 and VAL also reversed the increased collagen fiber (MTC) staining observed in CH group rats as measured by quantitation of %MTC area. Sections from Control group rats demonstrated a normal distribution of collagen fibers between cardiac muscles (Fig. 4I, i), while sections from CH group rats exhibited extensive fibrotic changes (Fig. 4I, ii) that were significantly reversed by VAL and ZCZ696 (both *p* < 0.001) (Fig. 4I, iii). However, collagen fibers were more prominent in the CH + VAL group than in the LCZ696 group, indicating a more robust antifibrotic effect of combined treatment (Fig. 4I, iv).

### LCZ696 and VAL ameliorated CH-associated cardiac histopathology

**Figure 5:**
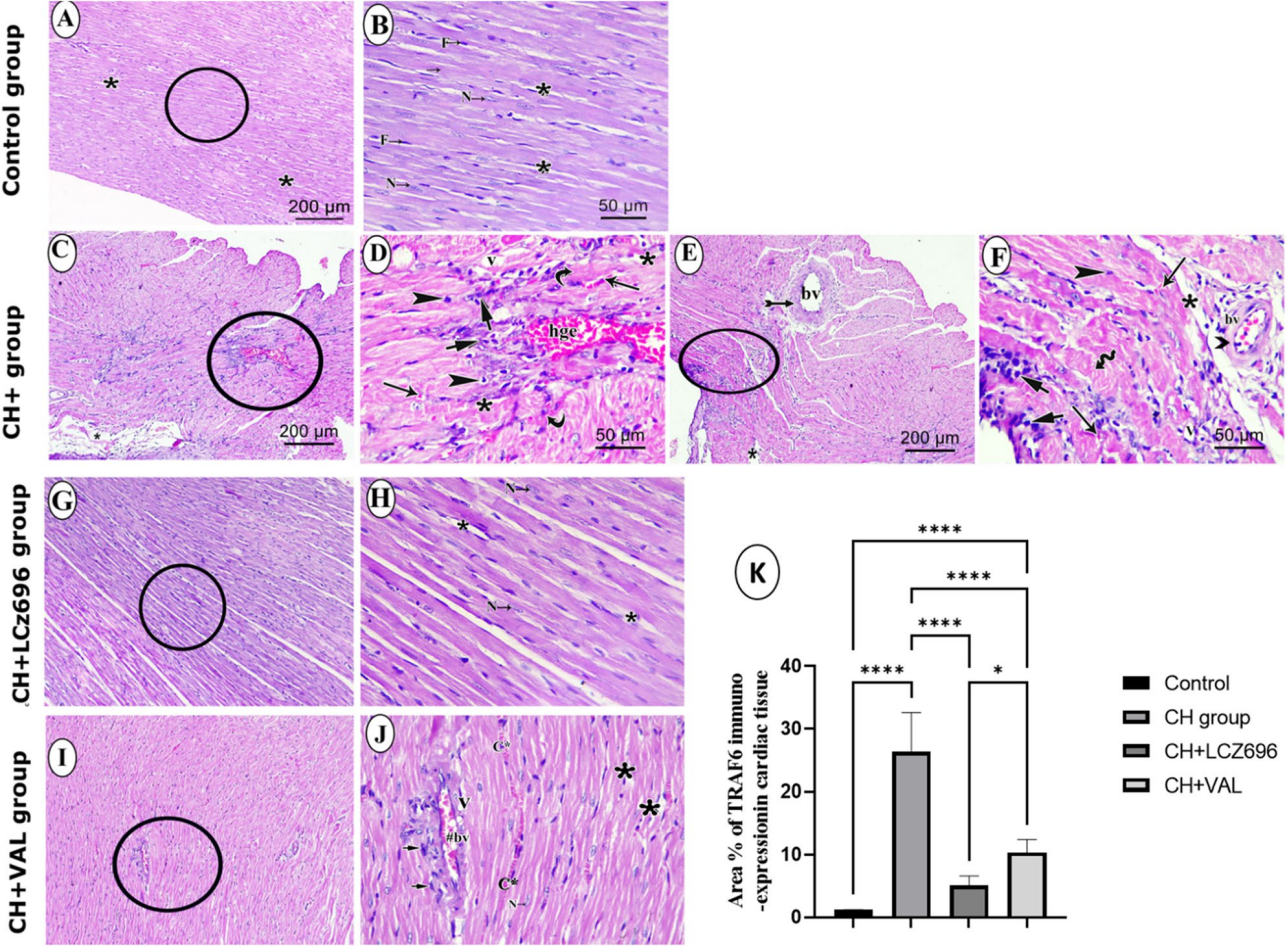
Microscopic images of H&E stain of cardiac muscle of left ventricle from different experimental groups. Control group (**A**, **B**): (**A**) shows normal myocardial architecture as branching and anatomizing muscle fibers with narrow interstitial spaces (*) and without inflammatory infiltrate or congestion. (**B**) Higher power of encircled area of (**A**) shows cardiomyocytes with acidophilic sarcoplasm, central oval vesicular nucleus (N), narrow interstitial spaces (*), and nucleus of interstitial fibroblast (F). CH group (**C**–**F**): (**C**) shows disturbed normal architecture of myocardial fibers and widening of interstitial spaces (*). (**D**) The higher power of encircled area of (**C**) shows abundant cellular infiltration (short arrow), pyknotic nuclei (arrowhead), areas of interstitial hemorrhage (hge), extravasated red blood cells (long arrow), wide interstitial spaces (*), dark acidophilic sarcoplasm (curved arrow) and areas of vacuolation (v). (**E**) another image of the CH group shows disturbed normal architecture of myocardial fibers, thickened wall (bifid arrow) of the blood vessel (bv), and widening of interstitial spaces (*). (**F**) Higher power of encircled area of (**E**) shows wavy myocardial fibers (wavy arrow), thickened wall of the blood vessel (** >**), abundant cellular infiltration (short arrow), extravasated red blood cells (long arrow), wide interstitial spaces (*) and areas of vacuolation (v). CH + LCZ696 group (**G**, **H**): **(G)** shows normal branching and anastomosing myocardial fibers separated by slightly interstitial space. **(H)** Higher power of encircled area of (**G**) group reveals myocardial fibers with central and vesicular nuclei almost like the control group. CH + VAL group (**I**, **J**): (**I**) reveals normal branching and anastomosing myocardial fibers, congested blood vessel (#bv) and mild spacing between myocardial fibers (*). **(J)** Higher power of encircled area of (**I**) shows, congested blood vessel (#bv) surrounded by cellular infiltrations (arrow), wide interstitial space (*), small vacuoles (v), congested capillaries (c*), and normal myocardial fibers with normal vesicular nuclei (N). Scale bar: (**A**, **C**, **E**, **G**, **I**) 200 μm × 100; (**B**, **D**, **F**, **H**, **J**) 50 μm × 400. K. thickness of the left ventricle wall of the different experimental groups. Values are the mean of 6 rats per group ± S.E.M. Group means were compared by one-way analysis of variance (ANOVA) followed by Tukey post hoc tests for multiple comparisons.

HE staining of LV sections from Control group rats revealed the typical structure of healthy myocardium, with myocardial fibers anastomosing and branching in different directions and containing centrally located vesicular nuclei (Fig. 5A, B). In contrast, sections from CH group rats showed severely disturbed fiber architecture, abundant cellular infiltration, congested blood vessels, areas of hemorrhage, extravasation of red blood cells, wide gaps between adjacent fibers, and areas of vacuolation and dark acidophilic sarcoplasm (Fig. 5C–F). These signs of histopathology were substantially reduced by LCZ696, although some dilation of intercellular spaces remained (Fig. 5G, H). Similarly, sections from the valsartan-treated group also showed normally arranged fibers and centrally located vesicular nuclei; however, many histopathological changes were still present, including congested blood vessels, mild cellular infiltration, wider interstitial spaces, small vacuoles, and congested capillaries (Fig. 5J, I).Histomorphometric evaluation of thickness of the left ventricular wall of the different experimental groups were performed (Fig. 5H). The statistical analysis showed a significant increase in the thicknesses of the left ventricular wall in hyperthyroid group when compared with the control group. interestingly, LCZ696 group displayed a significant decrement in the thicknesses of left ventricular wall when compared to hyperthroid group. While in valsartan group, despite it revealed a significant decrease in ventricular wall thickness compared to hyperthroid group , it exhibted a significant difference from LCZ696 group.

### LCZ696 and VAL reversed CH-associated cardiac ultrastructural damage

**Figure 6:**
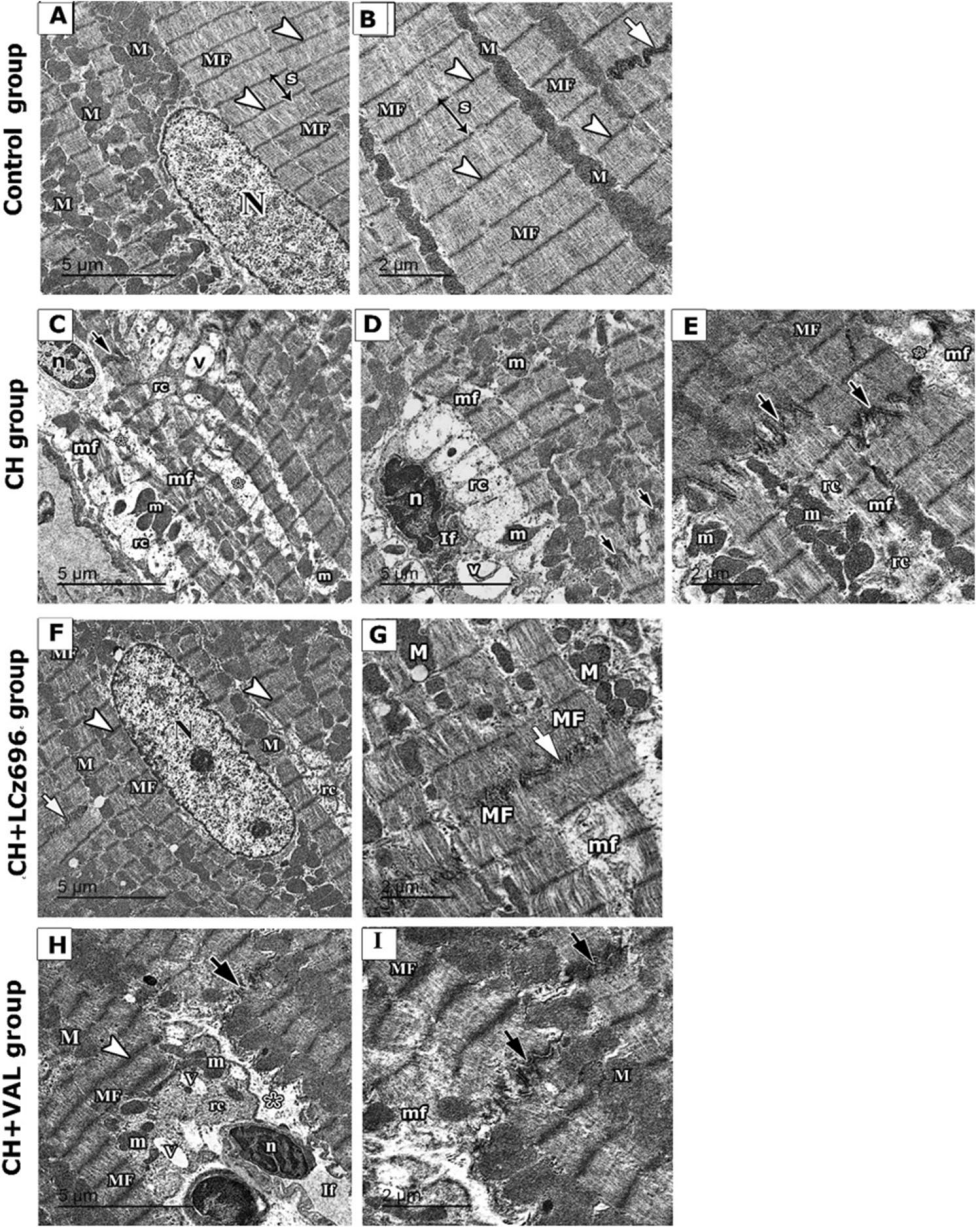
Electron micrographs of ultrathin sections in left ventricles of different experimental groups. (**A**, **B**) Control group showing myofibrils (MF) with narrow interfibrillar space. The oval euchromatic nucleus is seen in one fiber (N). Regular arrays of myofibrils are closely arranged within sarcomere (s) from Z line to Z line (arrowhead). Abundant mitochondria (M) were typically intact and neatly arranged in rows between the myofibrils. An intact intercalated disc can be seen in between the myofibril (short white arrow). (**C**–**E**) CH group showing cardiomyocyte with waviness and disturbed myofibrils (mf) with rarefied cytoplasm (rc) and heterochromatic nucleus (n). Wide separation between the myofibrils (*), many distorted and irregularly arranged mitochondria of different sizes and shapes (m), and areas of cytoplasmic vacuolations (V) can be observed. The interstitial tissue space is widened with infiltrating cells (If) that have a heterochromatic elongated nucleus (n). A distorted intercalated disc (short black arrow) can be noticed. (**F**, **G**) CH + LCZ696 group exhibited cardiomyocytes with a euchromatic nucleus (N) and nucleolus, regular transverse striations of myofibrils (MF) with regular (Z) lines (arrowhead). Rows of nearly normal mitochondria (M) are regularly arranged in between the myofibrils (MF) with an intact intercalated disc (short white arrow) can be seen. Area of rarefied cytoplasm (rc) can be noticed. (**H**, **I**) CH + VAL group reveals that some areas show regular transverse striations of myofibrils (MF) with regular (Z) lines (arrowhead). Rows of nearly normal mitochondria (M) are regularly arranged in between the myofibrils (MF). Other areas reveal separation between the myofibrils with rarefied cytoplasm (rc), some distorted and irregularly arranged mitochondria of different sizes and shapes (m), and areas of cytoplasmic vacuolations (V). The interstitial tissue space is widened with infiltrating cells (If) that have a heterochromatic irregular nucleus (n). A distorted intercalated disc (short black arrow) can be noticed.

Electron microphotographs of LV sections from Control group rats showed cardiac cells of typical morphology containing regular myofibril arrays closely arranged within sarcomeres (from Z line to Z line) and abundant mitochondria arranged in loose rows between myofibrils (Fig. 6A, B). In contrast, sections from CH group rats displayed more scattered myofibrils within sarcomeres (from Z line to Z line), larger interfibrillar spaces, dense pyknotic mitochondria of different shapes, patchy areas of cytoplasmic spaces, altered Z lines, and disruption of intercalated disks, heterochromatic and shrunken myocyte nuclei, and inflammatory cell infiltration (Fig. 6C–E). These ultrastructural abnormalities were largely reversed by LCZ696 (Fig. 6F, G). Sections from valsartan-treated rats also showed less severe abnormalities in cardiomyocyte and sarcomere structure compared to the CH group, but some signs of pathology remained, including atypically shaped mitochondria, and patchy areas of cytoplasmic spaces, altered Z lines, and disruption of intercalated disks (Fig. 6H, I).

## Discussion

Hyperthyroidism is one of the most common endocrinal diseases, and myocardial hypertrophy is a late but frequently lethal outcome^5^. Ge et al.^15^ reported that LCZ696 substantially reduced hyperthyroidism-associated cardiac remodeling, especially hypertrophy. The present study was designed both to validate the therapeutic potential of LCZ696 for the treatment of hyperthyroidism-induced cardiac hypertrophy and elucidate the possible underlying molecular mechanisms, including the contributions of *let-7b* and *miR-377* miRNAs. We found that LCZ696 was generally more efficacious against cardiac hypertrophy, inflammation, fibrosis, and disruption of autophagy than valsartan alone as evidenced by greater normalization of associated markers. Further, we present evidence that these therapeutic effects are associated with superior normalization of *let-7b* and *miR-377* expression.

Rats exposed to daily T4 treatment for 4 weeks exhibited hyperthyroid (HT) status as manifested by increased serum free T3 and T4 concentrations as well as marked weight loss. This experimentally induced hyperthyroidism rapidly induced cardiomyopathy as evidenced by marked increases in HR, SBP, and LVESP, and further examination revealed multiple signs indicative of myocardial hypertrophy, including greater dry heart/body weight ratio^16^, elevated serum concentrations of NT-proBNP and FGF-23, myocardial upregulation of the remodeling marker MMP-9, collagen fiber accumulation^17^, and upregulation of various fibrotic marker genes and the regulatory miRNA *let-7b*^18^. It is well documented that increased TH levels enhance HR and cardiac output through activation of the SNS and RAS among other mechanisms^19^, leading to myocardial hypertrophy^5^. All changes in marker expression indicative of hyperthyroid-associated cardiac hypertrophy were partially reversed by VAL alone and to greater degrees by LCZ696.

Myocardial hypertrophy is a progressive condition initiated and driven by chronic inflammation and ensuing fibrosis^20^. Our study revealed an increased inflammatory response in the hyperthyroid model as evidenced by serum elevations of proinflammatory cytokines (TNF-α, IL-1β, and IL-6) as well as upregulated mRNA expression of the stress-response transcription factor *NF-kβ*, and the TNF-α signal transduction protein TRF6 in LV sections from the CH group. Treatment with SAC and/or VAL significantly reduced the inflammatory response as indicated by reversal of these marker changes, consistent with previous studies reporting that LCZ696 ameliorated diabetic cardiomyopathy by inhibiting cardiac inflammatory and oxidative stress^21^. Further, LCZ696 induced substantial improvements beyond those produced by valsartan alone^22^.

MicroRNAs (miRNAs) are short non-coding sequences comprising 21–23 nucleotides^6^ that regulate gene expression at the post-transcriptional level by binding to the 3’-untranslated regions (3’-UTRs) of target mRNAs, thereby blocking protein translation and promoting mRNA degradation^23^. Several miRNAs are strongly implicated in the pathogenesis of cardiac hypertrophy, including the autophagic pathway regulator *miR-377*^7,24^. Autophagy is a cellular recycling pathway that conserves both cellular energy and nutrient supply under stress^25^. In this study, the cardiac hypertrophy group showed marked upregulation of *miR-377*^7,24^, which was reversed by LCZ696 and valsartan. The cardiac hypertrophy group also showed marked downregulation of the autophagic markers *LC-3* and *Beclin* but upregulation of *p62* and *mTOR*^10,26^. These changes were also reversed by valsartan and LCZ696^27^, possibly by normalizing *miR-377* expression and causing a rebound increase in autophagy^7,24^. In turn, upregulation of autophagy by these agents could reduce pathological cardiac remodeling by enhancing energy balance, nutrient reserves, and amino acid reserves^16^. Consistent with this notion, the CH group also exhibited significant downregulation of the myocardial metabolic regulator *PPAR-ϒ*^17^, and this decrease was similarly reversed by valsartan and to a greater extent by LCZ696. A previous study reported that *PPAR-ϒ* knockout induced cardiac hypertrophy^28^, strongly suggesting that normalization of expression levels by LCZ696 is a central therapeutic mechanism underlying the reversal of hypertrophy.

There are three major forms of autophagy, macroautophagy, microautophagy, and chaperone-mediated autophagy^18^. Macroautophagy is the most studied type and includes a mitochondrial-specific sub-type (so-called mitophagy) that is indispensable for reversing mitochondrial insufficiency and conserving cellular functionality in models of heart ischemia/reperfusion and for improving ventricular vitality under metabolite starvation by maintaining ATP and amino acid levels^19^. In turn, sustained metabolism can reduce cardiac remodeling, including fibrosis, ventricular dilatation, and hypertrophy. Suppression of autophagy will lead to the accumulation of potentially cytotoxic unfolded and misfolded proteins, as well as to increased cytoplasmic p62.

Interesetingly, The autophagy pathway collaborates with the ubiquitin-proteasomal system for the degradation of the unfolded and miss-folded proteins to abrogate the unfolded protein response (UPR) and the subsequent inflammation and apoptosis for conserving cellular vitality and the positive energy balance^20^. LC3 and beclin are the elements of the autophagy pathway, they are responsible for autophagosome membrane formation and nucleation for the damaged organelles and unfolded protein cargo to be lysed with the lysosomes and recycled to maintain cellular integrity^29,30^. However, the massive accumulation of the unfolded and misfolded proteins over the capacity of the cellular ubiquitin-proteasomal/autophagy system as these systems could deal with the small protein aggregates^31^. The abovementioned consequences lead to cardiac cells exhaustion and propagation of the endoplasmic stress signaling pathway (ER stress) which inhibits or switches off cardiac autophagy and promotes P62 accumulation that activates both cardiac inflammation, apoptosis, and subsequent remodeling^32^.

Collectively, these proteins can aggravate cardiac oxidative stress, inflammation, and apoptosis as well as upregulate the expression of the TGF-β/SMAD /fibrotic pathway, further worsening cardiac remodeling and functional insufficiency^28,33^. The results of the current study are consistent with the notion that cardiac hypertrophy is preceded by sharp upregulation in the mRNA expression levels of profibrotic markers (*TGF-β, SMAD-2, SMAD-3, SMAD-4*, and *NF-κβ*) and marked downregulation of antifibrotic *SMAD-7* mRNA and *let-7b* miRNA. All of these changes were normalized by LCZ696 and valsartan. The upregulation of *let-7b* by LCZ696 and valsartan illustrates a novel antifibrotic effect of these drugs through epigenetic regulation of fibrogenesis. Further, the efficacy of sacubitril/valsartan for reversal of *let-7b* downregulation was superior to valsartan alone, which may explain the superior efficacy of the combination for suppressing cardiac remodeling^34^. Histopathological findings and ultrastructural examination also support the greater efficacy of LCZ696 compared to valsartan, as LCZ696 eliminated most abnormalities observed in the CH group, while many signs of pathology remained following valsartan treatment, including myofibril disorganization, dense pyknotic mitochondria, and disruption of intercalated disks.

Based on the findings of the current study and previous results, we speculate that LCZ696 is more effective at ameliorating hyperthyroidism-induced cardiac hypertrophy than valsartan alone due to stronger modulation of *miR-377* and *let-7b*, miRNAs indispensable for the regulation of cardiac autophagy and fibrotic pathways, respectively.

## Materials and methods

### Animals

All surgical procedures and protocols were performed per the *Guide for the Care and Use of Laboratory Animals *(*US NIH Publication NO.85-23, revised 1996)* and approved by the *Zagazig University Institutional Animal Care Unit Committee, Zagazig University, Zagazig, Egypt* (approval number ZU-IACUC/3/H/2021)*.* All animal results are reported following ARRIVE guidelines.

Eight-week-old male Wistar rats weighing 200–220 g were obtained from the Faculty of Veterinary Medicine, Zagazig University, Egypt. Rats were housed in plastic cages (3 rats/cage) under a controlled environment (24 °C, 55% ± 5% relative humidity, 12/12-h light/dark cycle) with free access to standard rat chow and tap water throughout the experimental period at the Faculty of Veterinary Medicine, Zagazig University. The animals were allowed 2 weeks for acclimatization before experiments. All animals were studied concurrently and at the same age.

### Drugs and chemicals

Levothyroxine (T4) and valsartan were purchased from Sigma-Aldrich (St. Louis, MO, USA) and LCZ696 (Sacubitril/valsartan) (Entresto®) from Novartis Pharma AG (Basel, Switzerland). Valsartan and LCZ696 were emulsified in corn oil (2 mg/kg) and administered to animals once daily by gastric lavage (p.o.). All other chemicals and reagents were of the highest analytical grade commercially available.

### Experimental groups and design

The thyrotoxicosis rat model was induced by daily intraperitoneal (i.p.) injection of levothyroxine (T4, 100 µg/kg/day) for 28 days^35^.

Sixty adult male Wistar rats were randomly divided into four equal groups of 15: (I) a Control group receiving corn oil vehicle (2 mL/kg/day; p.o.), (II) a cardiac hyperthyroid (CH) group receiving T4 (100 µg/kg/day, i.p.) plus corn oil (2 mL/kg/day, p.o.), (III) a cardiac hyperthyroid plus sacubitril/valsartan (CH + LCZ696) group receiving T4 (100 µg/kg/day, i.p.) for 28 days followed by LCZ696 (60 mg/kg/day) for 14 days, and (IV) a cardiac hyperthyroid plus valsartan group (CH + VAL) receiving T4 (100 µg/kg/day, i.p.) for 28 days^15^ followed by valsartan (30 mg/kg/day) for 14 days^13^.

At the end of the treatment period, animals were weighed using an electronic balance (BXX 40; BOECO, Germany) and then anesthetized. Hemodynamic parameters were measured under anesthesia using the Power Lab data acquisition and analysis system (AD Instruments, Australia). The thorax was then opened and the heart rapidly removed, weighed, and processed for molecular, histological, or biochemical assessments as described.

### Measurement of hemodynamic parameters

Rats were anesthetized by i.p. injection of ketamine 90 mg/kg and xylazine 10 mg/kg. For measuring systolic blood pressure (SBP), the right carotid artery was exposed and cannulated with a PE-50 catheter connected to a blood pressure transducer and amplifier (FE224Quad Bridge Amp). The catheter was advanced into the left ventricle (LV) for recording the left ventricular systolic pressure (LVSP, mmHg). Pressure data were recorded for 10 min. Heart rate (HR) was recorded using three-needle electrodes fixed subcutaneously in the right arm (black lead), left arm (red lead), and left leg (green lead) and connected via an electrocardiography limb cable to an amplifier (FE136 Animal Bio, AD Instruments). All amplified data were recorded and analyzed using Power Lab (AD Instruments, Australia).

### Blood sampling

After hemodynamic measurements, blood samples (about 4–5 mL/rat) were collected from the cannulated right carotid artery by insertion of a PE-50 catheter and allowed to clot for 2 h at room temperature. The serum was obtained by centrifugation at 3000 rpm for 15 min and stored at − 20 °C for subsequent measurements of THs, pro-B-type natriuretic peptide (NT-proBNP), and fibroblast growth factor (FGF)-23.

### Preparation of cardiac tissue for histopathology

After hemodynamic measurements and blood sampling, the thorax was opened and cardiac arrest was induced by injecting 14 mM potassium chloride. The heart was then quickly removed, washed with saline, dried on filter paper, and weighed using a BXX 40 analytic scale (BOECO, Germany). Cardiac hypertrophy was evaluated by the heart weight (g) to body weight (g) ratio.

Subsequently, the LV was dissected and divided into four cross-sections. The first section (apex) was fixed in 10% neutral buffered formalin (pH, 7.4) for subsequent histopathology, the second in 4% glutaraldehyde for transmission electron microscopy (TEM), the third was collected in QIAzol (Qiagen, Hilden, Germany) at 50 mg/mL and stored at − 80 °C for later total RNA extraction and gene expression analysis, and the fourth was snap-frozen in liquid nitrogen then stored at − 80 °C for subsequent biochemical analyses.

### Measurements of serum hormones and markers of cardiac hypertrophy, fibrosis, and inflammation

Free thyroxine FT4, triiodothyronine FT3, pro-B-type natriuretic peptide (NT-proBNP), and FGF-23 were measured in serum using rat enzyme-linked immunosorbent assay kits (thyroxine FT4: CUSABIO, Wuhan, China, code CSB-E05079r; triiodothyronine FT3: NT-proBNP: MyBioSource, San Diego, CA, USA, Cat# MBS704791; FGF23: MyBioSource, San Diego, CA, USA, Cat# MBS2515950) according to the respective manufacturer's instructions. Serum concentrations of the inflammatory cytokines IL-1β, TNF-α, and IL-6 were measured using rat quantitative ELISA kits (all from R&D Systems Inc., Minneapolis, MN, USA).

### Evaluation of miRNA and mRNA expression levels in cardiac tissue

Total RNA was extracted from 50 mg of the dissected LV with 1 mL QIAzol (Qiagen) according to the manufacturer’s instructions and assayed for concentration and quality by measuring the absorbance at 260 and 280 nm using a NanoDrop® ND-1000 UV–Vis Spectrophotometer (Thermo Scientific, Waltham, MA, USA). Samples with an OD 260/280 ratio between 1.8 and 2.0 were considered of sufficient quality for subsequent miRNA and mRNA expression analyses. For measurement of miRNAs, a 50 ng sample of total extracted RNA was reverse transcribed to cDNA in a 20-µL reaction volume consisting of 5 µL nuclease-free water, 4 µL of 5× miRCURY RT reaction buffer, 2.5 µL of 10× miRCURY RT Enzyme Mix, 1.2 µL of a predesigned stem-loop primer (Table 1) (the primer for miRNA was designed via online software for designing stem-loop primers http://www.srnaprimerdb.com from the miRNA mature sequence that collected from the miRNA database https://www.mirbase.org/), and 10 µL of RNase-free water. Reverse transcription was induced by incubation at 42 °C for 60 min and was followed by enzyme inactivation at 95 °C according to the manufacturer’s instructions (Qiagen, Germany). Samples of cDNA were then stored at − 20 °C until PCR measured of miRNA expression. Alternatively, cDNA was prepared from mRNA using a high capacity reverse transcriptase kit (Applied Biosystem, Foster City, CS, USA) in a final reaction volume of 20 µL containing 10 µL master mix, 10 µL RNA sample, and 1 µg total RNA. The recovered cDNA was diluted 1:10 in nucleases free water and stored in aliquots at − 20 °C for subsequent quantitative (q)PCR using the Rotor-Gene Q 2 plex real-time thermal cycler (Qiagen, Germany) according to previously described methods^36,37^. In brief, the 20-µL reaction volume included 10 µL TOPreal syberGreen (Enzynomics, Korea), 1 µL each of forward and reverse primer (synthesized by Sangon Biotech, Beijing, China; see Table 1 for sequences), 1 µL of 1:10 diluted cDNA, and nuclease-free water up to 20 µL. The thermocycler conditions were initial denaturation at 95 °C for 10 min, 40 cycles of denaturation at 95 °C for 10 s, annealing at 60 °C for 15 s, and extension at 72 °C for 15 s, followed by melt curve analysis. Messenger RNA expression was measured as the fold-change relative to *Gapdh* mRNA expression, while miRNA expression was measured using U6 as the internal control according to previously reported methods^38^. Briefly, Δct was calculated as the ct difference between the target gene and reference gene expression, then ΔΔ ct was calculated as the difference between Δct of the sample and the average Δct of the control. Finally, the fold-change in gene expression was calculated as 2^−(ΔΔ ct)^.

**Table 1:** Primer sequences.

Gene	Forward primer 3′– > 5′	Reverse primer 3′– > 5′	Pb	Accession no
Gapdh	GCATCTTCTTGTGCAGTGCC	TACGGCCAAATCCGTTCACA	74	NM_017008.4
Beclin-1	GAATGGAGGGGTCTAAGGCG	CTTCCTCCTGGCTCTCTCT	180	NM_001034117.1
LC-3	GAAATGGTCACCCCACGAGT	ACACAGTTTTCCCATGCCCA	147	NM_012823.2
mTOR	GCAATGGGCACGAGTTTGTT	AGTGTGTTCACCAGGCCAAA	94	NM_019906.2
P62	GGAAGCTGAAACATGGGCAC	CCAAGGGTCCACCTGAACAA	183	NM_181550.2
PPAR-ϒ	GAGATCCTCCTGTTGACCCAG	CCACAGAGCTGATTCCGAAGT	129	NM_013124.3
BNP	CAGAAGCTGCTGGAGCTGATA	TCCGGTCTATCTTCTGCCCA	196	NM_031545.1
ANB	CCTGGACTGGGGAAGTCAAC	ATCTATCGGAGGGGTCCCAG	78	NM_012612.2
Smad-2	CAAACGTGCACAGGTGACAG	GACTGGCGTTGGAAGAAGGA	83	NM_001277450.1
Smad-3	CTGGGCAAGTTCTCCAGAGTT	AAGGGCAGGATGGACGACAT	148	NM_013095.3
Smad-4	CTTGGGGCAAGACTGCAAAC	GGTACAGTCAATGCGTCCCA	124	NM_019275.3
NF-κβ	ATTAGCCAGCGCATCCAGAC	ATCTTGAGCTCGGCAGTGTT	200	NM_199267.2
Smad-7	GAGTCTCGGAGGAAGAGGCT	CTGCTCGCATAAGCTGCTGG	84	NM_030858.2
TGF-β1	AGGGCTACCATGCCAACTTC	CCACGTAGTAGACGATGGGC	168	NM_021578.2

Let-7 b	Stem loop	GTCGTATCCAGTGCAGGGTCCGAGGTATTCGCACTGGATACGACAACCAC		
	Forward	AACACGCTGAGGTAGTAGGTT		
	Reverse	GTCGTATCCAGTGCAGGGT		
miR-377	Stem loop	GTCGTATCCAGTGCAGGGTCCGAGGTATTCGCACTGGATACGACATCAAC		
	Forward	AACAATCGGCGTCATGCAG		
	Reverse	GTCGTATCCAGTGCAGGGT		
U-6	Stem loop	AACGCTTCACGAATTTGCGTG		
	Forward	GCTCGCTTCGGCAGCACA		
	Reverse	GAGGTATTCGCACCAGAGGA		

### Histological and ultrastructure examination

Portions of dissected LV from five rats per treatment group were fixed in 10% neutral formalin at room temperature for 48–72 h, dehydrated in an increasing alcohol series, immersed in benzene for 20 min, embedded in paraffin, and cut into 5-µm thick sections. Sections were deparaffinized in xylene and stained with hematoxylin and eosin (HE) to assess histopathological changes or with Masson’s trichrome (MTC) stain to identify regions of interstitial fibrosis using a Nex ES Special Stainer (Ventana Medical Systems). Sections were then examined by light microscopy^39^. The thickness of of the left ventricular wall were evaluated in 10 non-overlapping low-powered microscope fields from 5 rats per experimental group using the Leica Qwin 500 image analyzer.

Other portions of the LV from five rats per group were fixed in 4% glutaraldehyde for 24–48 h, washed in phosphate buffer (pH 7.2–7.4), post-fixed in a buffered solution of 1% osmium tetroxide for 2 h, and washed four times in the same buffer without osmium tetroxide (20 min/wash). Fixed samples were dehydrated in an increasing ethanol gradient (30%, 50%, 70%, 90%, and 100%), cleared by two immersions in propylene oxide, and embedded in Epon resin. Ultrathin sections (60–90 nm) were cut and placed on copper grids for staining with lead citrate and uranyl acetate. Sections were then examined at the electron microscopy unit of Mansoura University using a Zeiss EM 100 S TEM with electron energy set to 60 kV^40^.

### Immunohistochemical staining

Immunohistochemical staining was performed using the avidin–biotin peroxidase complex method. Briefly, formalin-fixed sections were incubated in 3% hydrogen peroxide: methanol (1:1) for 30 min at room temperature to quench endogenous peroxidase activity, heated for 20 min in 10 mM citrate buffer (pH 6.0) for antigen retrieval, cooled at room temperature for 20 min, then treated with one or more of the following primary antibodies at 4 °C overnight: MMP-9 antibody (rabbit polyclonal, 1:250; cat. no. ab38898, Abcam, Cambridge, MA, USA), LC3-III antibody (rabbit polyclonal, 1:4000; cat. no. NB600-1384, Novus Biologicals, Cambridge, UK), p62/SQSTM1 antibody (mouse monoclonal, 1:40,000, cat. no. WH0008878M1, Sigma-Aldrich, Buchs, Switzerland) and/or TRAF6 antibody (rabbit polyclonal, 20 µg/mL; cat. no. PA5-119468, Invitrogen, Waltham, MA, USA). Sections were washed twice for 5 min in phosphate-buffered saline (PBS) then incubated sequentially with the appropriate secondary antibody and then with peroxidase-conjugated streptavidin diluted 1:3000 in PBS for 15 min. Immunolabeling was visualized by the addition of 0.02% 3,30 diaminobenzidine tetrahydrochloride. Sections were then counterstained with hematoxylin, dehydrated in gradient ethanol, and mounted in Canada balsam^36,41^.

### Quantitation of immunostaining and MTC staining for autophagy and fibrosis

The thickeThe mean fractional areas (% areas) of MMP-9-, TRAF-6-, LC3-, and P62-positive immunoreactivity and collagen fiber disposition were assessed in 10 non-overlapping high-powered microscope fields from 5 rats per experimental group using the Leica Qwin 500 image analyzer (Microsystems Imaging Solutions Ltd, Cambridge, United Kingdom). All analyses were conducted at the image-analyzing unit, Anatomy Department, Faculty of Medicine, Zagazig, University, Egypt.

### Statistical analysis

All datasets were tested for normality before group comparisons and are expressed as mean ± standard error of the mean. Group means were compared by one-way analysis of variance (ANOVA) followed by Tukey post hoc tests for multiple comparisons. All statistical calculations were performed using GraphPad Prism 9 (GraphPad Software, San Diego, CA). A *p* < 0.05 was considered significant for all tests.

## Supplementary Information

Supplementary Information 1.

Supplementary Information 2.

## Data Availability

The datasets generated and/or analysed during the current study are available in the supplementary material.
